# Correlation Analysis of Body Muscle-Fat Composition Parameters and CT Severity Index with Disease Severity in Acute Pancreatitis

**DOI:** 10.5152/tjg.2026.25489

**Published:** 2026-03-16

**Authors:** WenYang Wu, YongBao Li

**Affiliations:** Department of Radiology, Hangzhou Yuhang District Second People’s Hospital, Zhejiang, China

**Keywords:** Abdominal CT, acute pancreatitis, muscle-fat composition, modified CT severity index, prognosis

## Abstract

**Background/Aims:**

To investigate the correlation between body muscle-fat composition parameters (visceral adipose tissue (VAT) area, total adipose tissue (TAT) area, skeletal muscle index (SMI)) and the modified computed tomography severity index (MCTSI) with acute pancreatitis (AP) severity and their predictive value for adverse outcomes.

**Materials and Methods:**

Two hundred thirty-six AP patients were enrolled. Patients were stratified by severity into 84 mild AP (MAP), 90 moderately severe AP (MSAP), and 62 severe AP (SAP), and were further grouped by 30-day survival (172 survivors, 64 non-survivors). Demographic data, laboratory parameters, and abdominal computed tomography (CT) data were collected. Intergroup differences were analyzed, predictive performance was assessed, and independent risk factors were identified.

**Results:**

The scores of VAT, TAT, VAT/TAT, VAT/SAT, and MCTSI progressively increased with the severity of AP (from MAP to MSAP and SAP). No significant difference was observed in SAT among the groups. Non-survivors had higher VAT, SAT, TAT, and MCTSI scores but lower SMI than survivors (all *P* < .05). Receiver-operating characteristic (ROC) analysis demonstrated prognostic value for VAT (area under the curve (AUC) = 0.823), TAT (AUC = 0.789), SMI (AUC = 0.778), and MCTSI (AUC = 0.819), with combined assessment achieving an AUC of 0.960. Multivariate analysis identified APACHE II score, HDL, VAT, TAT, SMI, and MCTSI as independent predictors (*P* < .05).

**Conclusion:**

Body muscle-fat composition parameters and CT severity index correlate strongly with AP severity and independently predict adverse outcomes. Combined use enhances predictive accuracy, offering valuable insights for risk stratification and individualized treatment.

**Main Points**

- Visceral adipose tissue (VAT), total adipose tissue (TAT), and modified computed tomography severity index (MCTSI) increase with acute pancreatitis (AP) severity, while skeletal muscle index (SMI) decreases, correlating strongly with disease progression.
- Combined assessment of muscle-fat parameters and MCTSI improves prognostic accuracy for adverse outcomes.
- Visceral adipose tissue (VAT), TAT, SMI, and MCTSI are independent predictors, facilitating early risk stratification and personalized treatment in AP.

## Introduction

Acute pancreatitis (AP) is an inflammatory condition of the pancreas marked by elevated pancreatic enzyme and severe abdominal pain. Its incidence is rising, making it a common clinical issue.^1^ AP is classified into mild (MAP), moderately severe (MSAP), and severe AP (SAP) forms. While MAP generally has a good prognosis, severe cases can lead to infected necrosis and multiple organ failure.^2^ Nevertheless, in the absence of timely diagnosis and intervention, 20%-35% of patients may develop SAP, a life-threatening condition associated with high morbidity, multi-organ dysfunction, and a mortality rate of up to 30%.^3^^,4^ Despite ongoing revisions to clinical guidelines, accurately assessing the severity and predicting the prognosis of AP remain significant clinical challenges. Conventional evaluation systems predominantly depend on clinical manifestations, laboratory markers, and isolated imaging features, yet often fail to provide a dynamic quantification of metabolic status and pathophysiological progression. This limitation can result in the underestimation of disease severity or delayed intervention. Consequently, there is an urgent need to identify comprehensive indicators that effectively reflect disease severity and predict adverse outcomes.

With advancements in medical imaging, computed tomography (CT) has become increasingly crucial in the diagnosis of AP. Abdominal CT not only demonstrates the extent of inflammation, necrosis, and associated complications^5^ but also offers quantitative anatomical data pertinent to metabolic status. Recent research has underscored the potential of CT-derived fat and muscle parameters, such as visceral adipose tissue (VAT), total adipose tissue (TAT), and skeletal muscle index (SMI), which reflect body fat distribution and muscle mass.^6^^,^^7^ These parameters provide valuable quantitative anatomical insights related to metabolic status, further emphasizing the utility of CT in this context.^8^^,^^9^ In AP patients, excessive visceral fat may exacerbate pancreatic injury through pro-inflammatory cytokine release, while abnormal skeletal muscle mass may indicate metabolic dysregulation or uncontrolled stress responses, both influencing disease progression and prognosis.^10^ However, systematic studies on the correlation between muscle-fat composition parameters and AP severity or outcomes are lacking, warranting further validation.

In 2004, Mortele et al.^11^ modified and simplified the CT severity index (CTSI) into the modified CT severity index (MCTSI) by including assessments of extrapancreatic organ involvement and simplifying necrosis evaluation. The MCTSI is a standardized scoring system for inflammation and necrosis, linked to AP severity, mortality, and treatment decisions.^12^ It effectively predicts severe AP, pancreatic necrosis, and organ failure.^13^ However, it may miss anatomical changes and local complications.^14^ Combining muscle-fat composition parameters with CT severity indices can provide a more comprehensive assessment, enhancing the understanding of AP and guiding personalized treatment.

Against this background, this study aims to quantitatively analyze muscle-fat composition parameters (including VAT, subcutaneous adipose tissue (SAT), TAT, fat distribution ratios, and SMI) and CT severity indices in AP patients using abdominal CT examinations. The study aimed to investigate their associations with disease severity and evaluate their predictive value for adverse outcomes. The findings may provide novel quantitative indicators for AP severity assessment, enhance risk stratification, and inform personalized therapeutic strategies.

## Materials and Methods

### Study Population

This single-center prospective study enrolled 236 patients with AP admitted between January 2023 and December 2024.

Inclusion Criteria: (1) Patients meeting the diagnostic criteria for AP as defined by the Guidelines for diagnosis and treatment of acute pancreatitis in China (2021): persistent epigastric pain; serum amylase and/or lipase levels ≥3 times the upper limit of normal; and imaging findings consistent with AP. Diagnosis required at least 2 of these criteria. (2) Patients aged ≥18 years. (3) Patients with first-onset disease who had not received any prior treatment or intervention before enrollment. (4) Patients who underwent contrast-enhanced abdominal CT within 48 hours of admission, with qualified image quality (no significant artifacts, such as metal interference or motion artifacts) and clearly visible lesions.

Exclusion Criteria: (1) Patients with symptom onset-to-admission interval >72 hours or those who had received treatment at other hospitals. (2) Patients with contraindications to CT. (3) Patients with malignancies, chronic liver or kidney failure, autoimmune diseases, pregnancy, or concurrent infections at other sites. (4) Patients with psychiatric disorders. (5) Patients who had used immunosuppressants within 6 months or had severe immunosuppression. (6) Patients with incomplete clinical data, lost to follow-up, or voluntary discharge.

All participants provided written informed consent, and the study was approved by the Ethics Committee of Hangzhou Yuhang District Second People’s Hospital (approval No.202101YH-1; approval date: January 12, 2021).

### Data Collection

Patient demographic and clinical data were collected and analyzed, including gender, age, body mass index (BMI), smoking status, alcohol consumption, etiology (non-biliary or biliary), medical history (hypertension, hyperlipidemia, or diabetes mellitus), Acute Physiology and Chronic Health Evaluation II (APACHE II) score, triglycerides (TG), total cholesterol (TC), high-density lipoprotein cholesterol (HDL-C), and low-density lipoprotein cholesterol (LDL-C) levels were evaluated.

### Grouping Criteria

According to the 2012 revised Atlanta Classification criteria,^15^ patients were stratified into 3 groups based on disease severity: MAP patients without organ dysfunction or local/systemic complications, typically recovering within 1-2 weeks with very low mortality; MSAP patients with transient organ dysfunction (≤48 hours) and/or local complications, having low early mortality unless complicated by infected necrosis which increases fatality; and SAP patients with persistent organ dysfunction (>48 hours) demonstrating high mortality rates. Additionally, patients were categorized into survival and non-survival groups based on 30-day outcomes.

### Abdominal Computed Tomography Examination

All patients underwent abdominal CT scanning using Siemens Somatom Sensation and GE 64-slice LightSpeed VCT scanners in the supine position with arms raised. Scanning parameters were set at a 120 kV tube voltage, 450 mA tube current, 512 × 512 matrix, and 5 mm slice thickness, covering from the pubic symphysis to the diaphragmatic dome. Non-contrast scans were performed first, followed by contrast-enhanced scans using non-ionic iodinated contrast medium (Ultravist) injected at a rate of 2.5-3.5 mL/s (total volume 80-100 mL), with arterial phase acquired at 30-35 s, portal phase at 60-70 s, and delayed phase at 160-180 s, all completed during a single breath-hold. Images were reconstructed and analyzed on a workstation. Quantitative CT bone density analysis software was used to measure tissue composition at the L3 vertebral level, with automatic segmentation and manual correction to differentiate tissues: skeletal muscle tissue (−29 to +150 HU), VAT (−150 to −50 HU), and SAT (−190 to −30 HU). Visceral adipose tissue (VAT) area, SAT area, TAT area (VAT + SAT), VAT/TAT ratio, VAT/SAT ratio, and SMI (normalized to height in cm^2^/m^2^) were calculated. All measurements were performed by 1 radiologist and independently reviewed by 2 radiologists for consensus evaluation.

The MCTSI was assessed based on: pancreatic inflammation (0: normal; 2: parenchymal abnormalities with/without peripancreatic edema; 4: fluid collections or fat necrosis), pancreatic necrosis (0: none; 2: ≤30%; 4: >30%), and extrapancreatic complications (2: pleural effusion, ascites, vascular/gastrointestinal involvement). Total scores were graded as mild (0-2), moderate (4-6), or severe (8-10).

### Statistical Analysis

Statistical analysis was performed using Statistical Package for the Social Sciences, version 24.0 software (IBM SPSS Corp.; Armonk, NY, USA). The Shapiro—Wilk test was used to assess the normality of distribution. Normally distributed continuous variables are expressed as mean ± standard deviation and compared using the independent samples *t*-test, while non-normally distributed variables are presented as median (interquartile range) and analyzed with the Mann-Whitney *U*-test (for 2 groups) or the Kruskal–Wallis *H* test (for multiple groups). Categorical data are expressed as frequencies (percentages) and compared using the chi-square test. Receiver-operating characteristic (ROC) curves were constructed to calculate the area under the curve (AUC), cutoff values, sensitivity, and specificity to evaluate the predictive value of muscle-fat composition parameters and the modified CT severity index, both individually and in combination, for the severity of AP. Multivariate logistic regression analysis was performed to identify independent risk factors for severe AP. A *P*-value < .05 was considered statistically significant.

## Results

### Muscle-Fat Composition Parameters and Modified Computed Tomography Severity Index in Acute Pancreatitis Patients with Different Severity Grades

**Figure 1.**
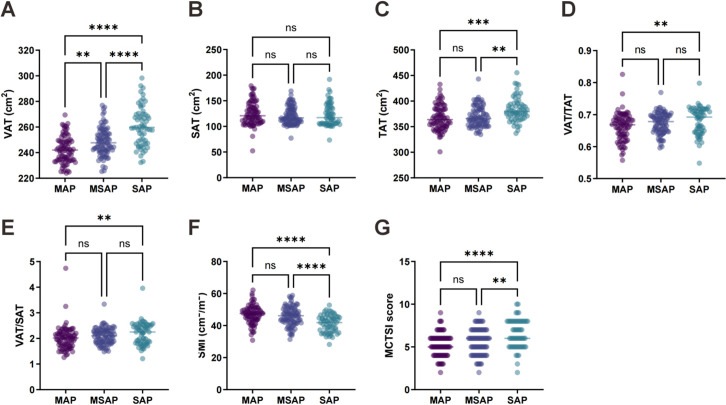
Comparison of muscle-fat composition parameters and modified computed tomography severity index (MCTSI) among 3 acute pancreatitis (AP) severity groups.

This study included 236 patients who were categorized by disease severity into the MAP (n = 84), MSAP (n = 90), and SAP (n = 62) groups. One-way ANOVA revealed significant differences in body muscle-fat composition parameters and CT severity indices among groups. Visceral adipose tissue (VAT) area demonstrated a progressive increase from MAP to MSAP to SAP (*P* < .001), while SAT area showed no significant intergroup differences. Total adipose tissue (TAT) area increased with disease severity (*P* < .001). Fat distribution ratios—VAT/TAT and VAT/SAT—exhibited increasing trends correlating with AP severity (both *P* < .05). Conversely, SMI decreased significantly among severity groups (*P* < .001). The MCTSI scores increased in parallel with disease severity (*P* < .001; Table 1, Figure 1). These findings indicate that worsening AP severity is associated with visceral fat accumulation, fat redistribution imbalance, skeletal muscle depletion, and elevated CT severity scores, suggesting the potential pathophysiological and prognostic relevance of muscle-fat parameters and imaging biomarkers.

### Comparison of Muscle-Fat Composition Parameters and Modified Computed Tomography Severity Index in Acute Pancreatitis Patients with Different Prognoses

**Figure 2.**
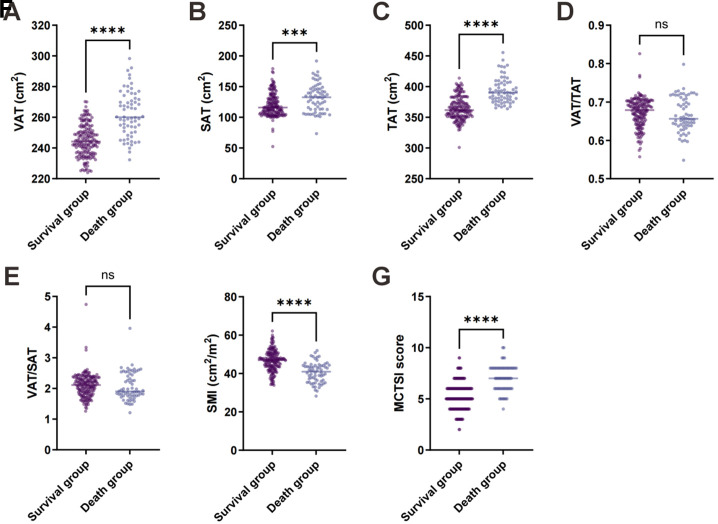
Comparison of muscle-fat composition parameters and modified computed tomography severity index (MCTSI) between acute pancreatitis (AP) patients with different outcomes.

The SAP patients were further stratified into survival (n = 172) and non-survival (n = 64) subgroups based on clinical outcomes. As shown in Table 2 and Figure 2, significant differences were observed between the survival and non-survival groups in the following parameters: VAT area, SAT area, TAT area, SMI, and MCTSI score. Specifically, the non-survival group exhibited significantly higher VAT, SAT, and TAT values but lower SMI compared to the survival group, along with elevated MCTSI scores. These findings suggested that non-survivors had more pronounced visceral fat accumulation, greater total fat content, reduced skeletal muscle mass, and higher CT severity indices. However, no significant differences were observed between the two groups in fat distribution ratios (VAT/TAT and VAT/SAT). These results showed that visceral fat accumulation, total fat content, skeletal muscle mass, and CTSI may be closely associated with adverse outcomes in AP patients, while fat distribution ratios may have limited prognostic value.

### Diagnostic Value of Muscle-Fat Composition Parameters and the Computed Tomography Severity Index for Prognosis in Severe Acute Pancreatitis

**Figure 3.**
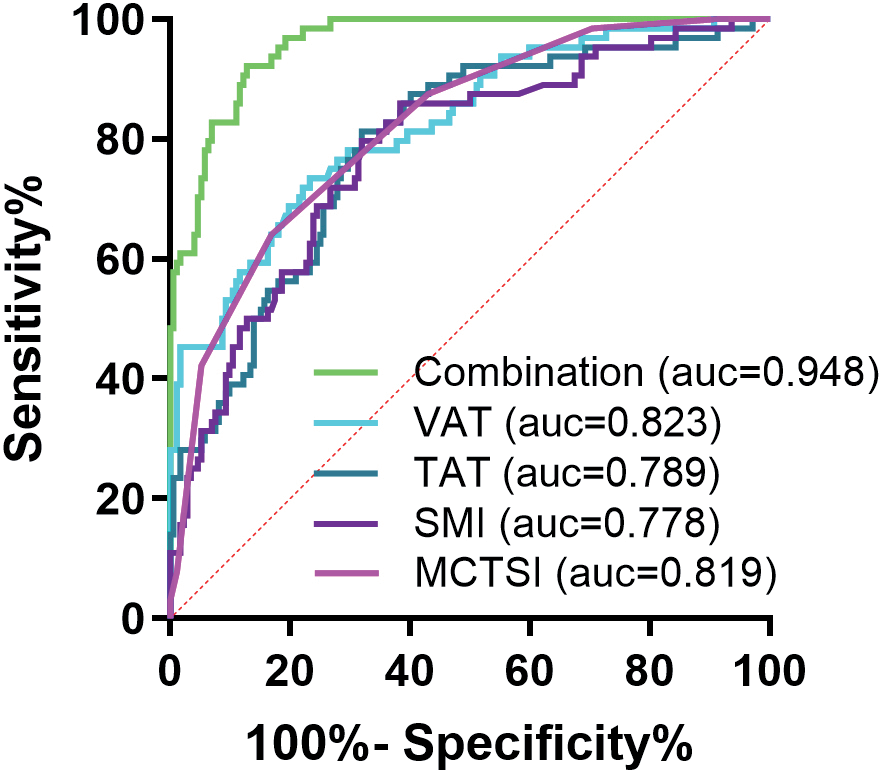
Receiver-operating characteristic (ROC) curves of muscle-fat composition parameters and modified computed tomography severity index (CTSI) for predicting prognosis in severe acute pancreatitis patients.

Receiver-operating characteristic (ROC) curve analysis was performed to evaluate the predictive value of muscle-fat composition parameters and CTSI for prognosis in SAP. The results demonstrated that VAT area showed an AUC of 0.823 (95% confidence interval [CI]: 0.762-0.884), with 73.44% sensitivity and 76.74% specificity at the optimal cutoff value of 252.9 cm^2^ (*P* < .001). Total adipose tissue (TAT) area yielded an AUC of 0.789 (95% CI: 0.724-0.854), with 81.25% sensitivity and 68.02% specificity at the cutoff of 372.5 cm^2^. Skeletal muscle index (SMI) achieved an AUC of 0.778 (95% CI: 0.712-0.845), showing 87.50% sensitivity and 61.63% specificity at 45.41 cm^2^/m^2^. The MCTSI score produced an AUC of 0.819 (95% CI: 0.762-0.878), with 64.06% sensitivity and 83.14% specificity at the cutoff of 6.50 points. Notably, the combined model (VAT + TAT + SMI + MCTSI score) significantly improved predictive performance (AUC = 0.960, 95% CI: 0.939-0.981), achieving 93.75% sensitivity and 81.98% specificity, indicating superior prognostic accuracy through multi-parameter analysis (Table 3, Figure 3).

### Multivariate Logistic Regression Analysis of Prognostic Factors in Acute Pancreatitis Patients

The comparison of baseline characteristics comparison (Table 4) revealed significant differences between non-survivors and survivors in APACHE II score, TG, and HDL levels, whereas no significant differences were observed in age, gender, BMI, smoking status, alcohol consumption, hypertension, diabetes mellitus, etiology, TC, or LDL levels. After adjusting for age and gender, multivariate analysis (Table 5) identified the following independent prognostic predictors: APACHE II score, HDL level, VAT area, TAT area, SMI, and MCTSI score.

## Discussion

AP is a serious digestive emergency with complex symptoms, making severity assessment and prognosis difficult, especially in severe cases where early risk identification is vital.^15^ This study examined the correlation between CT-derived muscle-fat parameters (VAT, TAT, SMI) and the MCTSI with AP severity and prognosis. Findings showed that VAT, TAT, and MCTSI scores increased with disease severity, while fat distribution ratios (VAT/TAT, VAT/SAT) stayed constant. Non-survivors had higher VAT, TAT, and MCTSI scores than survivors. Multivariate logistic regression identified these parameters as independent predictors of poor outcomes, with their combined use enhancing predictive accuracy.

While numerous studies have linked BMI to AP severity,^16^ BMI alone fails to quantify fat distribution or fully explain the pathological mechanisms underlying AP progression. Obesity, characterized by abnormal fat accumulation, is increasingly recognized not only as an energy storage condition but also as an endocrine disorder.^17^^,^^18^ Adipose tissue secretes adipokines that modulate inflammation and lipid metabolism, influencing the release of cytokines such as tumor necrosis factor-α, interleukin-6, and adiponectin.^19^^-^^22^ The findings of elevated VAT and TAT with worsening disease severity, along with significantly higher values in non-survivors, supported this hypothesis. These findings are highly consistent with previous studies. A systematic review of 15 studies (2000-2022) reported significant associations between VAT and AP severity in 10 studies.^23^ Xie et al. demonstrated that VAT was significantly higher in SAP and MSAP groups compared to MAP (*P* < .001) and correlated strongly with severity scores (APACHE-II ≥ 8, Ranson ≥ 3, BISAP ≥ 3, SIRS ≥ 2). VAT also had the highest AUC (0.943, 95% CI: 0.909-0.976) among prognostic indicators, further validating VAT as an independent risk factor.^24^ Although SAT showed no intergroup differences, increased TAT suggested that overall fat accumulation may exacerbate pancreatic injury through metabolic disturbances, such as insulin resistance, lipotoxicity. VAT-derived adipokines promote pro-inflammatory mediator release, potentially contributing to systemic inflammatory responses and increasing susceptibility to AP in overweight or obese individuals.^25^^,^^26^ Additionally, the marked reduction in SMI among non-survivors may reflect protein catabolism during stress-induced malnutrition.^27^ Rapid nutritional decline in AP patients compromises immune defenses, increasing risks of infections, organ dysfunction, and mortality.^28^^,^^29^

The MCTSI, as a widely used imaging scoring system, effectively demonstrates pancreatic necrosis, inflammatory changes, and extrapancreatic complications in AP patients, providing a comprehensive assessment of disease severity and prognostic prediction.^13^^,^^30^ A study by Alberti P et al.^13^ found that CT-based indices outperformed the classic APACHE-II score in evaluating severity parameters of AP. Consistent with this, the present study showed that MCTSI scores were significantly elevated in non-survivors, with each 1-point increase associated with approximately a 3.04-fold higher mortality risk (odds ratio = 3.04, 95% CI: 1.98-4.67). Although in the study by Tahir H et al.,^30^ the AUC of MCTSI for predicting AP severity was only 0.645 with low specificity, the MCTSI enabled accurate risk stratification for SAP, assisting clinicians in evaluating disease progression and adjusting treatment strategies to improve clinical outcomes.^27^ Notably, this study demonstrated that combining muscle-fat parameters (VAT, TAT, SMI) with MCTSI scores enhanced the predictive model’s performance (AUC = 0.960, 95% CI: 0.939-0.981), with sensitivity and specificity reaching 93.75% and 81.98% respectively, significantly outperforming individual parameters. This suggested that muscle-fat metabolic abnormalities and imaging-documented injuries may jointly influence AP prognosis through distinct pathways, with combined evaluation better capturing disease complexity.

This study provided novel quantitative tools for AP risk stratification, addressing limitations of traditional assessments that rely on clinical manifestations and laboratory markers, which may be delayed or confounded by therapeutic interventions. In contrast, abdominal CT, routinely performed early after admission, simultaneously provides both anatomical and metabolic information with superior objectivity and timeliness. The integration of VAT, TAT, SMI, and MCTSI enables comprehensive multidimensional evaluation within 48 hours of admission, facilitating early identification of high-risk patients for personalized management strategies. Specifically, patients with markedly elevated VAT or MCTSI scores may benefit from aggressive organ support, while those with abnormal SMI may require targeted nutritional interventions. The combined model demonstrates near-excellent predictive performance (AUC > 0.9), suggesting potential for development into clinical decision-support systems enabling dynamic monitoring of disease progression.

Several limitations warrant consideration. As a single-center prospective study, the generalizability of findings requires validation through multicenter studies. Although CT measurements followed standardized protocols, potential variations across imaging equipment or operators necessitate the establishment of unified scanning and post-processing protocols. Furthermore, the baseline data collected in this study were limited, and dynamic monitoring data were not incorporated. Consequently, the progression of muscle-fat parameters throughout the disease and their impact on prognosis remain unclear. Furthermore, while major confounders were adjusted, additional factors including medication history and nutritional support strategies merit further investigation to fully elucidate their prognostic significance.

In conclusion, CT-derived body composition parameters and severity indices demonstrate significant potential for enhancing severity assessment and prognosis prediction in AP. Systematic investigation of these quantitative indicators may yield novel approaches for clinical management, advancing the implementation of precision medicine in AP care to ultimately improve patient outcomes and quality of life.

## Figures and Tables

**Table 1.** Comparison of Muscle-Fat Composition Parameters and Modified CT Severity Index According to the Severity of AP

**Parameter**	**MAP Group (n = 84)**	**MSAP Group (n = 90)**	**SAP Group (n = 62)**	*P* **-value**
VAT (cm^2^)	242.31 ± 10.39	248.59 ± 10.81	260.77 ± 15.25	< .001
SAT (cm^2^)	121.26 (107.59, 138.58)	116.47 (10.36, 133.47)	177.18 (105.56, 136.90)	.505
TAT (cm^2^)	365.18 (350.87, 383.85)	366.32 (353.67, 383.77)	379.76 (367.06, 394.15)	< .001
VAT/TAT ratio	0.669 (0.631,0.690)	0.679 (0.650,0.689)	0.692 (0.646, 0.717)	.003
VAT/SAT ratio	2.01 (1.71, 2.22)	2.09 (1.86, 2.31)	2.25 (1.83, 2.53)	.002
SMI (cm^2^/m^2^)	46.91 ± 5.67	45.68 ± 5.89	41.34 ± 5.34	< .001
MCTSI score	5 (4, 6)	6 (4, 7)	6 (5, 8)	< .001

CT, Computed tomography; AP, acute pancreatitis; VAT, visceral adipose tissue; SAT, subcutaneous adipose tissue; TAT, total adipose tissue; SMI, skeletal muscle index; MCTSI, modified CT severity index; MAP, mild AP; MSAP, moderately severe AP; SAP, severe AP.

**Table 2.** Comparison of Muscle-Fat Composition Parameters and Modified CT Severity Index in AP Patients with Different Outcomes

**Parameter**	**Survival Group (n = 172)**	**Non-survival Group (n = 64)**	*P* **-value**
VAT (cm^2^)	244.89 ± 10.37	362.08 ± 14.76	< .001
SAT (cm^2^)	115.89 (105.22, 130.81)	133.1 (112.93, 146.30)	< .001
TAT (cm^2^)	361.89 (351.43, 376.06)	390.65 (378.28, 406.47)	< .001
VAT/TAT ratio	0.679 (0.649, 0.700)	0.657 (0.638, 0.712)	.362
VAT/SAT ratio	2.11 (1.85, 2.34)	1.91 (1.76, 2.48)	.356
SMI (cm^2^/m^2^)	46.63 ± 5.50	40.53 ± 5.29	< .001
MCTSI score	5 (4, 6)	7 (6, 8)	< .001

CT, Computed tomography; AP, acute pancreatitis; VAT, visceral adipose tissue; SAT, subcutaneous adipose tissue; TAT, total adipose tissue; SMI, skeletal muscle index; MCTSI, modified CT severity index.

**Table 3.** ROC Analysis of Muscle-Fat Composition Parameters and CT Severity Index for Predicting Prognosis in Severe Acute Pancreatitis Patients

**Parameter**	**AUC**	**95% CI**	**Cut-off value**	**Sensitivity (%)**	**Specificity (%)**	*P* **-value**
VAT (cm^2^)	0.823	0.762-0.884	252.9	73.44	76.74	< .001
TAT (cm^2^)	0.789	0.724-0.854	372.5	81.25	68.02	< .001
SMI (cm^2^/m^2^)	0.778	0.712-0.845	45.41	87.50	61.63	< .001
MCTSI score	0.819	0.762-0.878	6.50	64.06	83.14	< .001
Combined model	0.960	0.939-0.981		93.75	81.98	< .001

ROC, Receiver operating characteristic; CT, computed tomography; VAT, visceral adipose tissue; TAT, total adipose tissue; SMI, skeletal muscle index; MCTSI, modified CT severity index; AUC, area under the curve.

**Table 4.** Baseline Characteristics of Acute Pancreatitis Patients by Outcome

**Factor**	**Survival group (n = 172)**	**Non-survival group (n = 64)**	*P* **-value**
Age (years)	58 (54, 63)	59 (53, 62)	.818
Gender			.768
Male	103 (59.88%)	37 (57.81%)	
Female	69 (40.12%)	27 (42.19%)	
BMI	23.01 ± 2.95	23.09 ± 3.22	
Smoking	55 (31.978%)	27 (42.19%)	.167
Alcohol use	60 (34.88%)	31 (48.44%)	.071
Hypertension	38 (22.09%)	18 (28.13%)	.390
Diabetes	51 (29.65%)	20 (31.25%)	.873
Etiology			.303
Biliary	74 (43.02%)	33 (51.56%)	
Non-biliary	98 (56.98%)	31 (48.44%)	
APACHE II score	12 (10, 14)	16 (14, 19)	< .001
Laboratory values			
TC (mmol/L)	6.32 ± 1.54	6.64 ± 1.65	.165
TG (mmol/L)	1.64 ± 0.27	1.95 ± 0.43	< .001
HDL (mmol/L)	0.84 (0.73, 0.92)	0.64 (0.57, 0.73)	< .001
LDL (mmol/L)	2.54 ± 0.43	2.43 ± 0.40	.076

BMI, Body mass index; APACHE II, Acute Physiology and Chronic Health Evaluation II; TC, total cholesterol; TG, triglycerides; HDL, high-density lipoprotein; LDL, low-density lipoprotein.

**Table 5.** Multivariate Logistic Regression Analysis of Prognostic Factors in Acute Pancreatitis Patients

**Variables**	β	**S.E**	**Z-value**	*P* **-value**	**OR (95% CI)**
Intercept	−95.49	30.54	−3.13	**.002**	0.00 (0.00-0.00)
Gender					
0					1.00 (Reference)
1	0.31	1.12	0.28	.783	1.36 (0.15-12.15)
Age	−0.03	0.08	−0.30	.765	0.98 (0.83-1.15)
APACHE II score	0.95	0.28	3.37	**< .001**	2.59 (1.49-4.50)
TG	1.33	1.58	0.84	.400	3.80 (0.17-84.76)
HDL	−10.60	3.98	−2.67	**.008**	0.00 (0.00-0.06)
VAT	0.16	0.06	2.81	**.005**	1.17 (1.05-1.31)
TAT	0.13	0.05	2.83	**.005**	1.14 (1.04-1.24)
SMI	−-0.20	0.09	−2.13	**.033**	0.82 (0.68-0.98)
MCTSI score	1.11	0.51	2.19	**.028**	3.04 (1.13-8.24)

APACHE II, Acute Physiology and Chronic Health Evaluation II; TG, triglycerides; HDL, high-density lipoprotein; VAT, visceral adipose tissue; TAT, total adipose tissue; SMI, skeletal muscle index; MCTSI, modified CT severity index. Note: A *P*-value < 0.05 was considered to be an independent factor affecting the prognosis of patients with acute pancreatitis.

## Data Availability

The data that support the findings of this study are available on request from the corresponding author.
